# Knockdown of phosphodiesterase 4D inhibits nasopharyngeal carcinoma proliferation via the epidermal growth factor receptor signaling pathway

**DOI:** 10.3892/ol.2014.2422

**Published:** 2014-08-08

**Authors:** TING XU, SIHAI WU, YUAN YUAN, GUOXIN YAN, DAJIANG XIAO

**Affiliations:** 1 Department of Otolaryngology, The Second People’s Hospital of Wuxi, Wuxi, Jiangsu 214002, P.R. China; 2 Department of Stomatology, The Second People’s Hospital of Wuxi, Wuxi, Jiangsu 214002, P.R. China

**Keywords:** phosphodiesterase 4D, nasopharyngeal carcinoma, epideramal growth factor receptor, proliferation

## Abstract

Phosphodiesterase 4D (PDE4D) is a subtype of metallohydrolases, and it has been reported that PDE4D functions as a proliferation promoting factor in certain types of cancer, including head and neck cancer. The present study first investigated the function of PDE4D in nasopharyngeal carcinoma (NPC). Western blot analysis was applied to detect PDE4D expression in NPC samples and cells. A lentiviral infection technique was used to stabilize the knockdown of PDE4D, which was subsequently examined *in vitro* and *in vivo*. The results showed that PDE4D was overexpressed in the NPC tissues and cells. Knockdown of PDE4D inhibited the growth of CNE2 and 5–8F, inducing cell cycle arrest in the G_0_/G_1_ phase in CNE2. These effects could be reversed by epidermal growth factor (EGF) stimulation. Furthermore, knockdown of PDE4D significantly inhibited the phosphorylation of epidermal growth factor receptor (EGFR) and AKT. The results were further validated in an NPC xenograft in nude mice. In conclusion, this study demonstrated that PDE4D may function as a proliferation promoting factor in NPC, by affecting the EGFR/PI3K/AKT signaling pathway. Therefore, the targeting of PDE4D may be a rational strategy in the treatment of NPC.

## Introduction

The phosphodiesterases (PDEs) are metallohydrolases that hydrolyze the secondary messengers, cyclic adenosine monophosphate (cAMP) or cyclic guanosine monophosphate (cGMP), into 5′-AMP or 5′-GMP, which is considered to be the only pathway to degrade cAMP and cGMP intracellularly (1). The impairment of cAMP or cGMP generation by the regulation of PDEs has been indicated in various cancer pathologies (2,3). The PDE subtype, PDE4D, belongs to the PDE4 subfamily, which includes the four isoforms, A, B, C and D. It has been demonstrated that PDE4D functions as a proliferation promoting factor in prostate cancer through the Sleeping Beauty transposon system (4). Pullamsetti *et al* (5) found that PDE4D induces human lung cancer proliferation *in vitro* and *in vivo* through the hypoxia-inducible transcription factor signaling pathway (5). Studies have also shown that PDE4D is involved in the transforming growth factor (TGF)-β1-induced epithelial-mesenchymal transition in the human alveolar epithelial type II A549 cell line. The upregulated PDE4D expression caused by TGF-β1 may contribute to the invasion and metastasis of A549 cells (6). Recently, Lin *et al* (7) found that PDE4D protein expression levels were elevated in multiple types of cancer, including head and neck cancer (HNC).

Nasopharyngeal carcinoma (NPC) is a type of HNC, however, due to unique clinical, etiological and biological characteristics, NPCs are distinct from other HNCs. NPC is one of the most common types of cancer in Southern China, particularly among those of Cantonese origin (8). Sothern China has the highest incidence of this disease, peaking at 50 cases per 100,000 individuals per year (9). Despite NPC being a generally radiosensitive disease, the presentation of local recurrence and distant metastasis is observed in certain patients following radiotherapy as a result of radioresistance (10). The development of therapies targeting the underlying molecular mechanisms of tumorigenesis has become a focus of attention, as the use of adjuvant chemotherapy has shown limited success.

Increasing evidence has shown that the overexpression of epidermal growth factor receptor (EGFR) is common in NPC; its signaling may be significant in the pathogenesis of NPC (11,12). A recent study has proposed EGFR as a novel target for NPC therapy (13). Epidermal growth factor (EGF) and TGF-α are natural ligands that bind to the extracellular domain of EGFR, subsequently activating EGFR and its downstream signaling proteins. This results in the modulation or activation of various cellular processes (14). In total, ~200 targets of the EGFR signaling pathway have been reported (15). One of the most significant signaling pathways is the phosphoinositide 3-kinase/AKT pathway. EGFR activates PI3K, which phosphorylates phosphatidylinositol 2-phosphate to phosphatidylinositol 3-phosphate, and subsequently activates AKT, as well as a number of its downstream effectors. This induces protein synthesis, cell growth and survival, proliferation, migration and angiogenesis (16). However, a direct association between the EGFR/PI3K/AKT axis and the PDE4D-cAMP axis in malignant cells has not been reported. In the present study, PDE4D was demonstrated to promote NPC cell proliferation *in vitro* and *in vivo*, which has been associated with the EGFR/PI3K/AKT signaling pathway.

## Patients and methods

### Patients and tissue preparation

In total, 40 fresh, poorly-differentiated NPC tissues and 21 normal nasopharyngeal epithelial tissues were obtained between January 2009 and December 2012 at the Department of Otolaryngology, The Second People’s Hospital of Wuxi (Wuxi, China). All patients had no history of previous malignancies, or treatment with radiotherapy or chemotherapy. The clinical stage of the patients was classified according to the Chinese 2008 NPC staging system (17). All samples were snap-frozen immediately and stored in liquid nitrogen prior to protein extraction. The study was approved by the Research Ethics Committee of the Second People’s Hosiptal of Wuxi (Wuxi, China) and written informed consent was obtained from all the patients. All specimens were handled and made anonymous according to the ethical and legal standards.

### Cell culture and lentiviral short hairpin (sh)RNA infection

The immortalized nasopharyngeal epithelial NP69 cells (Shanghai Bogoo Biological Technology Co., Ltd., Shanghai, China) were cultured in keratinocyte-SFM (Invitrogen Life Technologies, Carlsbad, CA, USA) supplemented with bovine pituitary extract (BD Biosciences, Franklin Lakes, NJ, USA). The human NPC cell lines, CNE1, CNE2, 5–8F, 6–10B and HNE1 (Shanghai Bogoo Biological Technology Co., Ltd., Shanghai, China), were cultured in Roswell Park Memorial Institute 1640 medium (Invitrogen Life Technologies, Carlsbad, CA, USA). PDE4D-targeted shRNA lentiviral particles (LV-PDE4D shRNA; sc-44004-v) and control shRNA lentiviral particles (ctr-shRNA; sc-108080) (both Santa Cruz Biotechnology, Inc., Santa Cruz, CA, USA) were infected into the CNE2 cells according to the manufacturer’s instructions. Following ≥14 days of selection using Puromycin (Invitrogen Life Technologies), the individual puromycin-resistant colonies were isolated and expanded.

### Cell viability assay and cell cycle analysis

The cells were plated in 96-well plates at a density of 2.5×10^3^ cells/well. Cell growth and viability was detected by MTT assay, as described previously (18). Briefly, the medium was replaced with fresh medium containing 5 mg/ml MTT reagent (Sigma-Aldrich, St. Louis, MO, USA) following various durations of culture (at 24, 36, 48 and 96 h). The cells were cultured for an additional 4 h at 37°C, then 100 μl dimethyl sulfoxide was added to each well to solubilize the colored crystals produced within the living cells. The absorbance was measured at a wavelength of 490 nm using a BioTek microplate reader (Bio-Rad, Hercules, CA, USA). In addition, cell cycle analysis was performed by flow cytometric analysis, as described previously (18). Briefly, the transfected cells were seeded onto a six well plate at a density of 105 cells/well in RPMI 1640 medium for 48 h. Next, the cells were harvested and fixed in ice cold 70% (v/v) ethanol for 15 min. The cells were then treated with RNase A (Sigma-Aldrich) and stained with 50 μg/ml propidium iodide (Abcam, Cambridge, UK), followed by incubation at 37°C for 30 min in the dark. Samples were analyzed using a FACScan flow cytometer (Becton Dickinson, Franklin Lakes, NJ, USA), according to the manufacturer’s instructions. In addition, the DNA content in the G1, S and G/M phases was analyzed using BD FACSDivaTM software (Becton Dickinson).

### Western blot analysis

Western blot analyses were performed as described previously (19). Briefly, a total of 50 μg protein from each sample was added to gel loading buffer (pH, 6.8; 125 mM 2× Tris-HCl; 4% SDS; 20% glycerol; 0.1% bromophenol blue; 2.5% β-mercaptoethanol), boiled for 5 min, separated by 10% SDS-polyacrylamide gel and then transferred onto polyvinylidene difluoride membranes. The blotted membranes were then incubated with primary polyclonal anti-PDE4D (sc-25814; 1:500), monoclonal anti-EGFR (sc-373746; 1:1,000), monoclonal anti -phosphorylated (p)-EGFR (sc-57543), monoclonal anti-AKT (sc-5298; 1:1,000) and polyclonal anti-pAKT (sc-33437; 1:300) antibodies, which were purchased from Santa Cruz Biotechnology, Inc., at 4°C overnight. After being washed with Tris-buffered saline with Tween 20 (SunShine Biotechnology Co., Ltd., Nanjing, China), the membranes were incubated with monoclonal horseradish peroxidase-conjuagted anti-rabbit or anti-mouse secondary antibodies for 1 h at room temperature. Protein bands were visualized using BeyoECL Plus Detection System (Beyotime Beyotime Institute of Biotechnology, Jiangsu, China). Protein expression levels were quantified using FluorChem FC2 and presented as the densitometric ratio of the targeted protein to β-actin. Cell protein lysate assays were performed in triplicate.

### Colony-forming assay

The cells were plated on six-well plates at 3×10^2^ cells per well and grown for 14 days. Next, the cells were washed twice with phosphate-buffered saline (SunShine Biotechnology Co., Ltd.), fixed with methanol/acetic acid (3:1; v/v: SunShine Biotechnology Co., Ltd.) and stained with 0.5% crystal violet (C3886; Sigma-Aldrich). The number of colonies was counted under a microscope (TE2000, Nikon Corp., Tokyo, Japan).

### EGF stimulation assay

CNE2 cells infected with ctr-shRNA or LV-PDE4D shRNA were treated with 100 ng/ml recombinant human EGF (AF-100-15; PerproTech, Rocky Hill, NJ, USA) for 2 h. Western blot analysis was used to detect protein levels at various treatment times (0, 10, 60 min and 6 h). The cell viability assay, cell cycle analysis and colony-forming assay were applied to detect the cell proliferation following EGF stimulation.

### Nude mice experiment

Five-week-old male BALB/c nude mice were purchased from the Institute of Laboratory Animal Sciences (Beijing, China). The CNE2 cells infected with LV-PDE4D shRNA and control (referred to as the LV-PDE4D shRNA and ctr-shRNA groups, respectively) were isolated. A total of 5×10^5^ cells of each type were injected subcutaneously into the dorsal flanks of two groups of nude mice, with each group containing five mice. Tumor size was measured every two days and the tumor volume was calculated as follows: Tumor volume (mm^3^) = (length × width^2^) × 0.5. Two weeks later, the mice were sacrificed using cervical dislocation and the tumors were dissected. The experiment was repeated three times. The use of animals in the present study complies with the Guide for the Care and Use of Laboratory Animals (National Research Council) (20). The study was approved by the Institutional Animal Care and Use Committee, Wuxi, Jiangsu, China.

### Immunohistochemistry

Immunohistochemistry was performed as described previously (19). Formalin-fixed, paraffin-embedded tissues of the transplanted tumors were sectioned to a 4-mm thickness and analyzed for anti-p-EGFR and anti-pAKT expression. Visualization was achieved using the EnVision peroxidase system (Dako, Carpinteria, CA, USA). The degree of immunostaining of the formalin-fixed, paraffin-embedded sections was reviewed and scored by two independent pathologists in a blinded manner. The percentage of positively-stained nuclear tumor cells was recorded. Scoring was determined according to the percentage of positive cells as follows: 1, ≤5% of cells; 2, 6–35% of cells; 3, 36–70% of cells; and 4, ≥71% of cells. An additional score was determined according to the intensity of staining as follows: 1, negative staining; 2, weak staining (light yellow); 3, moderate staining (yellowish brown); and 4, strong staining (brown) (20).

### Statistical analysis

Quantitative data are presented as the mean ± standard deviation. The statistical significance of the differences between the two groups was analyzed using a two-sided, unpaired Student’s t-test for equal variances or Welch’s corrected t-test for unequal variances. Statistical analysis was performed using SPSS 13.0 software (SPSS, Inc., Chicago, IL, USA). P<0.05 was considered to indicate a statistically significant difference.

## Results

### PDE4D is upregulated in clinical specimens and human NPC cell lines

**Figure 1:**
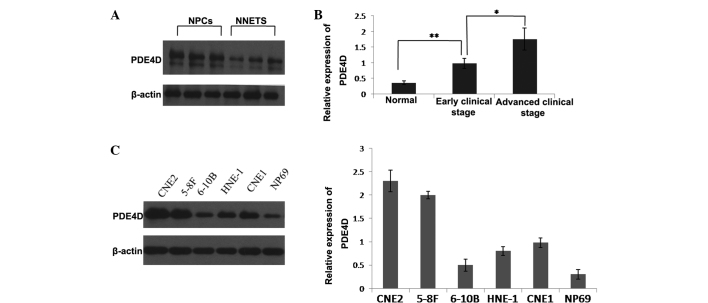
Expression of PDE4D detected in clinical samples and cell lines. (A) The expression of PDE4D was detected in 40 cases of NPC and 21 cases of NNET by western blot analysis. PDE4D expression was found to be significantly higher in the NPC cases when compared with NNET cases (P<0.05). (B) A correlation was identified between PDE4D and the clinical parameters of NPC. Early clinical stage NPC tissues exhibited higher PDE4D expression than normal tissues (P<0.01 vs. control). (C) Expression of PDE4D in five NPC cell lines and NP69. Each experiment was performed in triplicate and the mean value was calculated. ^*^P<0.05 and ^**^P<0.01. PDE4D, phosphodiesterase 4D; NNET, normal nasopharyngeal epithelial tissues; NPC, nasopharyngeal carcinoma.

The clinical significance of PDE4D was investigated in 40 human NPC samples. The results showed that PDE4D was significantly upregulated in the NPC samples compared with the normal nasopharynx epithelial samples (Fig. 1A). The upregulation of PDE4D expression was found to correlate with an advanced clinical stage of NPC (Fig. 1B). The expression of PDE4D was further examined in human NPC cell lines and the immortalized non-tumorigenic NP69 cell line. Western blot analysis showed that PDE4D was increased in all five NPC cell lines compared with the NP69 cell line. Among the five NPC cell lines, CNE2 and 5–8F showed relatively higher PDE4D expression levels, while 6–10B showed a relatively lower PDE4D expression level (Fig. 1C).

### Knockdown of PDE4D inhibits the growth of NPC cells

**Figure 2:**
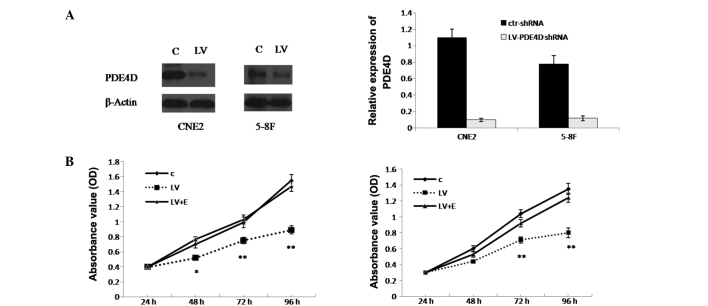
Knockdown of PDE4D inhibits the growth of NPC cells, which is reversed by EGF stimulation. (A) Western blot analysis results and graph presenting the infection efficiency of PDE4D-targeted shRNA lentiviral particles in CNE2 and 5–8F cells. Following infection, PDE4D expression was significantly inhibited in the two cell lines. (B) Effect of PDE4D-targeted shRNA lentiviral particles on cell proliferation measured by MTT assay following infection in CNE2 and 5–8F cells. The effect of EGF stimulation on the proliferation of the NPC cells, which were infected with PDE4D-targeted shRNA lentiviral particles, is also shown. ^*^P<0.05 and ^**^P<0.01 vs. C and LV+E. PDE4D, phosphodiesterase 4D; NPC, nasopharyngeal carcinoma; EGF, epidermal growth factor; shRNA, short hairpin RNA; OD, optical density; C, cells infected with control shRNA lentiviral particles; LV, cells infected with PDE4D-targeted shRNA lentiviral particles; LV+E, cells infected with PDE4D-targeted shRNA lentiviral particles followed by 100 ng/ml EGF treatment; ctr-shRNA, control shRNA lentiviral particles.

**Figure 3:**
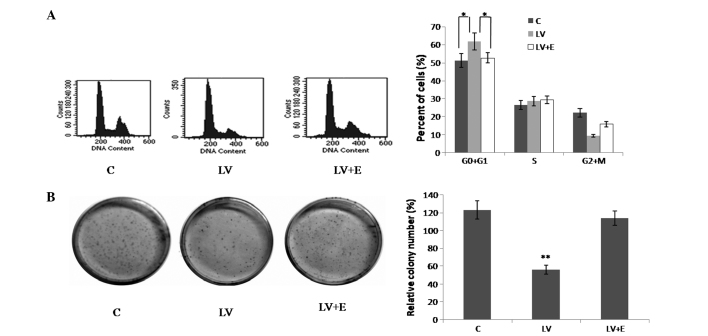
Effect of PDE4D on cell cycle arrest and colony formation in CNE2 cells. (A) Knockdown of PDE4D induced cell cycle arrest in the G_0_/G_1_ phase in the CNE2 cells, which was reversed by EGF stimulation. ^*^P<0.05 vs. C and LV+E. (B) Results of the colony formation assay showed that knockdown of PDE4D inhibited colony formation in the CNE2 cells, which was reversed by EGF stimulation. ^**^P<0.01 vs. C and LV+E. PDE4D, phosphodiesterase 4D; EGF, epidermal growth factor; shRNA, short hairpin RNA; C, cells infected with control shRNA lentiviral particles; LV, cells infected with PDE4D-targeted shRNA lentiviral particles; LV+E, cells infected with PDE4D-targeted shRNA lentiviral particles followed by 100 ng/ml EGF treatment.

CNE2 and 5–8F cells were selected for further study due to their relatively high PDE4D expression level. The results revealed that the infection efficiency of the CNE2 and 5–8F cells was 89.68±1.7 and 87.32±1.54%, respectively. In addition, the western blot analysis showed that PDE4D was successfully inhibited in the CNE2 and 5–8F cells (80.5±1.6 and 83.23±2.4%, respectively; P<0.01; Fig. 2A). To explore the effect of PDE4D on NPC cell growth, the MTT assay was applied to detect proliferation. It was demonstrated that CNE2 cell growth was inhibited by 36.3% (P<0.05), while 5–8F cell growth was inhibited by 29.7% (P<0.05) (Fig. 2B). As revealed in the colony formation assay, CNE2 cells infected with LV-PDE4D shRNA exhibited much smaller colonies compared with the control cells (Fig. 3B).

### Knockdown of PDE4D induces cell cycle arrest in the G_0_/G_1_ phase in CNE2 cells

Flow cytometry was performed to examine the CNE2 cell cycle. The results showed that compared with the control, the CNE2 cells infected with LV-PDE4D shRNA exhibited an increased percentage of cells in the G_0_/G_1_ phase and fewer cells in the G_2_/M phase (Fig. 3A; P<0.05).

### Knockdown of PDE4D inhibits the phosphorylation of EGFR and AKT

**Figure 4:**
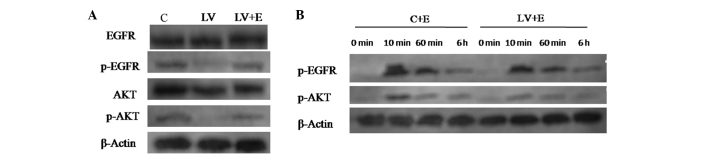
Western blot analysis of EGFR signaling pathway proteins of the CNE2 cells following infection with LV-PDE4D shRNA and EGF stimulation. (A) Knockdown of PDE4D inactivates EGFR and AKT in CNE2 cells, and EGF stimulation reverses the efficiency of LV-PDE4D shRNA. (B) Various time points following stimulation with 100 ng/ml of EGF. The phosphorylation of EGFR and AKT for C+E compared with LV+E cells. After 10 min, EGF was found to promote the phosphorylation of of EGFR and AKT, however, following PDE4D knockdown, EGF stimulation decreased. Each experiment was performed in triplicate and the mean value was calculated. PDE4D, phosphodiesterase 4D; NPC, nasopharyngeal carcinoma; EGFR, epidermal growth factor receptor; shRNA, short hairpin RNA; C, cells infected with control shRNA lentiviral particles; LV, cells infected with PDE4D-targeted shRNA lentiviral particles; LV+E, cells infected with PDE4D-targeted shRNA lentiviral particles followed by 100 ng/ml EGF treatment; C+E, control cells stimulated by 100 ng/ml EGF; p-EGFR, phosphorylated EGFR; p-AKT, phosphorylated AKT.

The expression of EGFR is common in NPC tissues and cell lines, and the EGFR/PI3K/AKT signaling pathway is important in the pathogenesis of NPC (20). However, the effect of PDE4D on the EGFR/PI3K/AKT signaling pathway remains unknown. The present study investigated whether the EGFR/PI3K/AKT axis is associated with the PDE4D-cAMP axis in CNE2 cells. p-EGFR and p-AKT were found to be expressed in the control CNE2 cells. Following the stable knockdown of PDE4D, p-EGFR and p-AKT levels were significantly decreased (Fig. 4A). Furthermore, following treatment by 100 ng/ml recombinant human EGF for 2 h, the CNE2 cells infected with LV-PDE4D shRNA showed increased phosphorylation of EGFR and AKT (Fig. 4A).

A further 100 ng/ml EGF was added to the CNE2 cells infected with ctr-shRNA and LV-PDE4D shRNA. At ~10 min, EGF began to promote the phosphorylation of EGFR and AKT. However, as time went by (0 min–6 h), the extent of EGFR and AKT phosphorylation was gradually weakened. The LV-PDE4D shRNA-infected CNE2 cells showed inferior phosphorylation of the two proteins compared with the control following EGF stimulation (Fig. 4B).

### EGF stimulation reverses the effect of LV-PDE4D shRNA on NPC cells

As demonstrated by MTT assay (Fig 2B), following treatment with 100 ng/ml recombinant human EGF, the LV-PDE4D shRNA-infected CNE2 and 5–8F cells exhibited increased cell proliferation compared with the untreated cells, which reversed the effect of LV-PDE4D shRNA on the NPC cells. Similar results were detected in the colony formation assay of the CNE2 cells (Fig. 3B).

### Knockdown of PDE4D suppresses the tumor growth of NPC cells in nude mice

**Figure 5:**
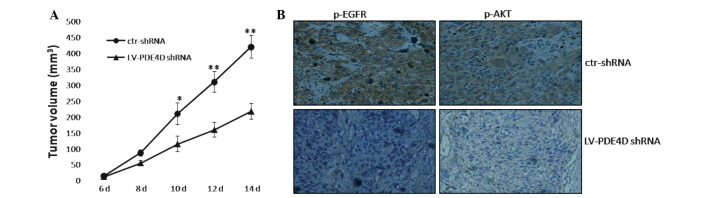
Knockdown of PDE4D inhibits tumor growth of NPC cells in nude mice. (A) CNE2 cells infected with LV-PDE4D shRNA and ctr-shRNA were injected subcutaneously into nude mice. At two weeks post-implantation, the LV-PDE4D shRNA-infected cells produced smaller tumors than those of the ctr-shRNA group. The growth curve represents the tumor volumes. Data are presented as the mean ± standard error of the mean of five mice. (B) At two weeks post-transplantation, the transplanted tumors of the two groups (each of five mice) were sectioned and stained for p-EGFR and p-AKT. Images were captured under inverted microscope at ×200 magnification. ^*^P<0.05 and ^**^P<0.01. shRNA, short hairpin RNA; p-EGFR, phosphorylated epidermal growth factor receptor; p-AKT, phosphorylated AKT; ctr-shRNA, control shRNA lentiviral particles.

The CNE2 cells that were infected with LV-PDE4D shRNA were injected subcutaneously into the dorsal flank of the nude mice. The tumor became palpable between six and seven days post-inoculation. All the mice developed tumors at the end of the experiment. At ~10 days post-implantation, a statistically significant difference was identified between the volume of the transplanted tumors in the two groups (P<0.05; Fig. 5A). At two weeks post-implantation, the mice injected with LV-PDE4D shRNA carried smaller burdens compared with the controls. The average tumor volume and weight of the LV-PDE4D shRNA-treated group were markedly reduced compared with the control group (P=0.023 and P=0.034, respectively). In addition, the staining intensity of p-EGFR and p-AKT in the tumor cells was significantly decreased compared with the control (P<0.05; Fig. 5B).

## Discussion

It has been reported that PDE4D functions as a proliferation-promoting factor in certain cancer types, including HNC (4–7). From genomic investigation, Lin *et al* (7) showed that the PDE4D gene was highly expressed in HNC. Analysis of the survival data from the Cancer Genome Atlas projects showed that high PDE4D mRNA expression levels are associated with a poor prognosis in HNC. However, there has been no further investigation on PDE4D in HNC. PDE4D was of great interest in the present study of NPC, as NPC is a unique type of HNC that has a high incidence in Southern China (8).

The present study first examined PDE4D in human NPC tissues and cells. The results revealed that PDE4D was significantly upregulated in the NPC samples and cells, which was found to correlate with an advanced clinical stage of NPC. These results are consistent with the study by Lin *et al* (7), which used immunohistochemical staining to show that PDE4D proteins are overexpressed in ovarian and endometrial tumors and melanomas, compared with corresponding non-transformed tissues (7). In the present study, for the expression of PDE4D in the NPC cells, the poorly-differentiated CNE2 cell line and the 5–8 F cell line with high metastatic potential exhibited relatively higher expression levels. While the highly-differentiated CNE1 cell line and the 6–10 cell line with poor metastatic potential exhibited relatively lower expression levels, which suggested that PDE4D may correlate with the differentiation and metastatic potential of the NPC cells. Subsequently, the CNE2 and 5–8 F cell lines were selected for further *in vitro* experiments.

To examine the role of PDE4D in the growth of NPC cells, LV-PDE4D-shRNA infection was used to knockdown PDE4D expression in the CNE2 and 5–8F cells. The results showed that the knockdown of PDE4D significantly inhibited the cell growth of the two cell lines. The cells were then treated with 100 ng/ml EGF, which reversed the cell proliferation inhibition induced by LV-PDE4D-shRNA. In addition, the colony formation assay showed a similar result in the CNE2 cells. The cell cycle assay further demonstrated that LV-PDE4D-shRNA inhibits CNE2 cell proliferation by inducing a G_0_/G_1_ arrest, which was also reversed by EGF stimulation. These results suggested that PDE4D may function by affecting the EGFR signal pathway in the growth of NPC cells.

The EGFR is commonly expressed in NPC cells and is one of the identified molecular targets for cancer treatment (23). In various NPC cell lines, the downregulation of EGFR by monoclonal antibodies and the use of specific pharmacological inhibitors have been shown to inhibit the EGFR signaling pathway and subsequently induce cell cycle arrest and cell proliferation suppression (24–26). However, the association between the EGFR signaling pathway and PDE4D has not been reported in cancer cells. In the present study, to further demonstrate the potential crosstalk existing between the PDE4D and EGFR/AKT axis, the EGFR signaling pathway was detected in the CNE2 cells by western blot analysis. It was demonstrated that following the knockdown of PDE4D in the CNE2 cells, the phosphorylation of EGFR and AKT were significantly inhibited. Following treatment with 100 ng/ml EGF, this inhibition was reversed. EGF-stimulated LV-PDE4D shRNA CNE2 cells showed inferior phosphorylation of EGFR and AKT compared with the control following EGF stimulation. Overall, the results suggested that the knockdown of PDE4D may suppress NPC cell growth through inhibition of the EGFR/AKT signaling pathway.

*In vivo* experiments further demonstrated that the knockdown of PDE4D suppressed tumorigenesis in a NPC murine xenograft model, suggesting that PDE4D may be a tumor-promoting factor in NPC. These results are consistent with the findings in melanoma, and breast and ovarian cancer (7). In order to demonstrate the crosstalk between PDE4D and the EGFR/PI3K/AKT axis, p-EGFR and p-AKT were examined in a nude mouse xenograft. The xenograft, which was injected with LV-PDE4D-shRNA-infected CNE2 cells, showed lower p-EGFR and p-AKT expression levels compared with the control xenograft. This suggested that PDE4D affects the EGFR/PI3K/AKT signaling pathway not only *in vitro,* but also *in vivo*, which is not affected by the internal environment.

Taken together, the present study demonstrated that the knockdown of PDE4D inhibits cell growth in NPC, partly by affecting the EGFR signal pathway, by suppressing the phosphorylation of EGFR and AKT. This suggests that PDE4D may contribute to the proliferation of NPC cells, and therefore, PDE4D is predicted to be a potential therapeutic target for the treatment of NPC.
